# Development of a GFP biosensor reporter for the unfolded protein response-signaling pathway in plants: incorporation of the bZIP60 intron into the GFP gene

**DOI:** 10.1080/15592324.2022.2098645

**Published:** 2022-07-20

**Authors:** Rina Carrillo, David A. Christopher

**Affiliations:** Department of Molecular Biosciences & Bioengineering, University of Hawaii, Honolulu, HI, USA

**Keywords:** endoplasmic reticulum stress, unfolded protein response

## Abstract

The ability to measure the activation of the unfolded protein response (UPR) in plants is important when they are exposed to stressful environments. To this end, we developed a unique and versatile biosensor-reporter system to indicate the activation of UPR in living plant cells. The small cytoplasmically spliced intron from the *bZIP60* locus was incorporated into the 5’ end of the GFP gene, creating the 35S::*bZIP60* intron:GFP construct. When this construct is transiently expressed in *Arabidopsis* protoplasts, the presence of the *bZIP60* intron prevents GFP mRNA from being translated under non-UPR conditions. However, when UPR is activated, the IRE1 kinase/ribonuclease splices this intron from the GFP mRNA and its translation proceeds, generating GFP fluorescence. We demonstrated the utility of the system in *Arabidopsis* leaf protoplasts treated with DTT, which is a chemical inducer of UPR, followed by visualization and quantification using confocal microscopy. The 35S::*bZIP60* intron:GFP construct was also expressed in protoplasts from an overexpressor line containing the coding sequence for the UPR-induced, protein folding chaperone, protein disulfide isomerase-9 (PDI9). PDI9 also influences the strength of the UPR signaling pathway. Protoplasts from WT and *PDI9* overexpressor plants treated with DTT exhibited significantly higher GFP fluorescence relative to untreated protoplasts, indicating that the *bZIP60* intron was spliced from the GFP mRNA in response to activation of UPR. RT-PCR further confirmed the higher induction of *PDI9* and *bZIP60* (total and spliced) mRNA levels in DTT-treated protoplasts relative to controls. This system can be adapted for monitoring crop stress and for basic studies dissecting the UPR signaling pathway.

## Introduction

During the course of a plant’s life cycle, it is exposed to changes and stresses in the environment that require it to sense and adapt to new conditions. A key cellular adaptation occurs in the endoplasmic reticulum (ER), which increases its capacity to synthesize, fold, and secrete proteins.^1^ When proteostasis is disrupted, such as upon exposure to heat stress,^2–4^ drought stress,^5,6^ and bacterial and viral infection,^7,8^ the accumulation of unfolded proteins exceeds the protein folding capacity of the ER, leading to ER-stress.^9,10^ In turn, the ER stress-signal transduction pathways invoke sensors and downstream transcription factors that activate the unfolded protein response (UPR,^11,12^). The UPR functions to restore protein homeostasis by inducing the cellular production of protein folding enzymes, such as protein disulfide isomerases (PDIs) and chaperones,^4^ while also temporarily down-regulating various metabolic processes,^13^ disaggregating unfolded protein complexes,^3^ or proteolytically degrading excess unfolded proteins (ERAD;^14–16^).

These adaptive processes highlight the role of the UPR and the associated sensor mechanisms that lead to downstream transcription factor cascades. One of the three different ER membrane-UPR sensors is the inositol requiring enzyme-1 (IRE1).^17^ The IRE1A/IRE1B complex is a ribonuclease-kinase^12,18^ that unconventionally splices the BASIC LEUCINE ZIPPER60 (*bZIP60*) mRNA to produce the active form of the nuclear-localized transcription factor, *bZIP60s*, which then activates the expression of downstream UPR genes.^11,19^ The IRE1A/IRE1B-mediated splicing of the *bZIP60* mRNA is enhanced at elevated growth temperatures and links UPR to heat stress.^20,21^

Because of the important role that the UPR plays in abiotic and biotic stress responses in plants,^4,17,21^ it is experimentally useful to develop reporter systems that can indicate when the UPR is activated, especially early in the pathway. Such a system can be utilized for monitoring crop stress and for studies dissecting the UPR signaling pathway in single cells and whole plants. Arabidopsis mesophyll protoplasts have previously been used to measure UPR induction via RT-PCR.^22^ In addition to PCR-based methods,^9,23^ other assays used to measure the UPR have also been described, including various phosphorylation assays that detect differences in the phosphorylation state of IRE1 as a proxy for UPR activation and therefore splicing of *bZIP60*.^23,24^ However, these methods can be labor-intensive, and the versality of a direct bZIP60 reporter as a marker for the UPR in real time is efficient and effective, and appropriately suited for the transient transfection system offered by *Arabidopsis* protoplasts.^25^

Recently, we developed an *in vivo* system for rapidly measuring protein–protein interactions during UPR in *Arabidopsis* protoplasts.^25^ Here, we describe a unique and versatile biosensor-reporter system to indicate the activation of UPR and ER stress in plants. It consists of incorporating the small cytoplasmically spliced intron from the *bZIP60* locus into the 5’ end of the GFP gene. This construct is then transiently expressed in Arabidopsis protoplasts. The presence of the *bZIP60* intron would prevent GFP mRNA from being translated under non-UPR conditions. However, upon unconventional splicing of the bZIP60 intron from the GFP mRNA, its translation can proceed, leading to GFP fluorescence. We demonstrate the working utility of the system in Arabidopsis protoplasts treated with DTT, which is a chemical inducer of UPR, followed by visualization and quantification using laser-scanning confocal microscopy and PCR. The use of this system can be expanded into transgenic crops and basic research deciphering the UPR pathway.

## Materials and methods

**Figure 1.**
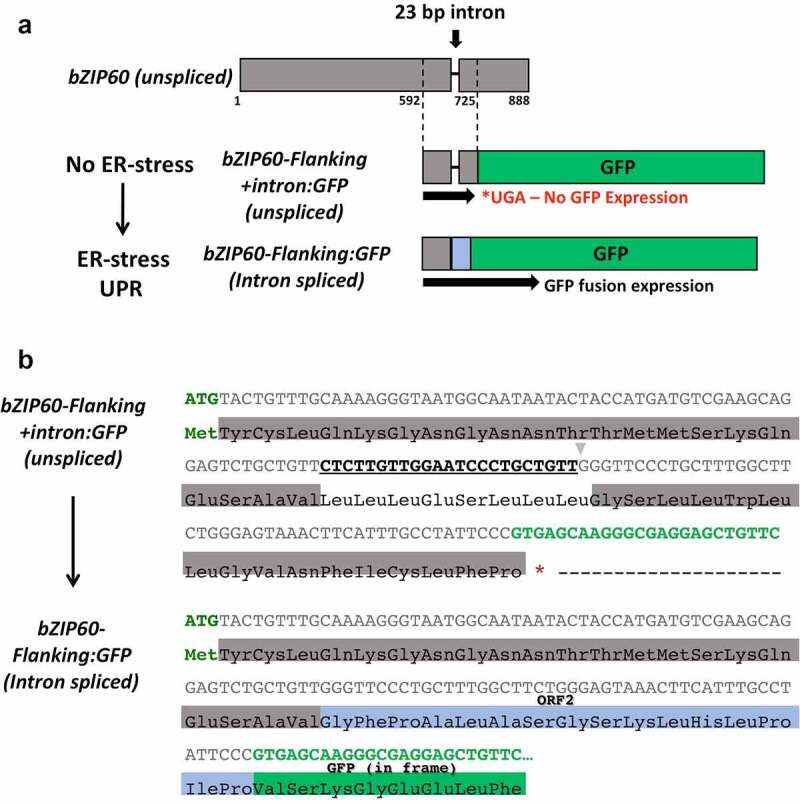
Construct design of the 35S::*bZIP60* intron:GFP reporter construct. a) The *Arabidopsis bZIP60* sequence flanking the IRE1-targeted 23-bp intron (592–725) was fused to GFP at the N-terminus. Translation of the unspliced sequence is interrupted by a stop codon (UGA, denoted by an *) after the *bZIP60* intron, resulting in no GFP expression. Thus, only splicing of *bZIP60* places GFP in frame with the *bZIP60* start codon, as a detection method for IRE1-mediated UPR activation. b) Nucleotide and corresponding protein sequences for both the unspliced (top) and spliced (bottom) variants of the 35S::*bZIP60* intron:GFP construct. The translation initiation codon ATG is shown in green text, *bZIP60* flanking exonic region in gray, 23-bp intronic region targeted by IRE1 is bolded and underlined, GFP coding region highlighted in bright green, and the translational stop codon is indicated by an *. Upon theoretical splicing of the 23-bp intron (CTCTTGTTGGAATCCCTGCTGTT), a frame-shift results in formation of ORF2 with the GFP coding sequence in-frame with the translation initiation codon. ORF2 has been documented as the natural endogenous frameshift that occurs in *bZIP60* mRNA,^11^ as indicated here highlighted in blue.

To create the 35S::*bZIP60* intron:GFP reporter construct, the 23 bp IRE1-targeted *bZIP60* intronic region (CTCTTGTTGGAATCCCTGCTGTT) was inserted upstream from the N-terminus of the GFP coding sequence as adapted from mammals.^26^ As a result, a stop codon (TGA) becomes in frame at the start of the GFP coding sequence (designated by an asterisk in Figure 1(b)). In addition, the exonic sequence flanking the *bZIP60* intronic region corresponding to nucleotide positions 592 through 725 (At1g42990.1) was also included to retain IRE1-specific splicing signals inherent within the transcript (Figure 1).^20^ The CaMV 35S promoter sequence was amplified from the previously described PDI9:mCherry-KDEL construct using primers engineered with KpnI and XhoI restriction sites and inserted between their respective sites in the pBluescript KS+ vector.^27^ The nos terminator was amplified from the previously described GFP (S65T) vector^28^ and inserted between NotI and SacI restriction sites in the developing plasmid. The GFP (S65T) construct (hereon referred to as GFP) is driven by the 35S promoter and previously reported to localize to the cytoplasm in *Arabidopsis* protoplasts.^28^ The bZIP60 intronic region fused to the coding sequence of GFP was designed using GeneWiz gene synthesis (GeneWiz, Inc., USA) with flanking XhoI and NotI restriction sites and inserted between their respective sites in the developing plasmid to yield the final construct, 35S::*bZIP60* intron:GFP, which was confirmed for accuracy by DNA sequencing (Figure 1). To confirm transfection efficiency between protoplast cells expressing varying fluorescence intensities of the 35S::*bZIP60* intron:GFP construct, cells were co-transfected with a 35S::mCherry control. For the generation of the 35S::mCherry control construct, a CaMV 35S promoter fragment amplified from pCAMBIA1302 with engineered KpnI and XhoI restriction sites was inserted within respective sites of the cloning vector, pBluescript KS+. The mCherry fragment was amplified from PDI9:mCherry-KDEL using primers engineered with XhoI and BamHI restriction sites and ligated into the plasmid template described above containing the 35S promoter. The 3’-UTR nos terminator sequence was amplified from the GFP construct described above and inserted between BamHI and NotI restriction sites to yield the final construct.

## Transient expression assay in *Arabidopsis* protoplasts and treatment with DTT

Protoplast isolation and transfection was performed using the Tape-*Arabidopsis* Sandwich protocol^29^ as further modified^30^ on 4-week-old *Arabidopsis* plants (WT Col and the PDI9 overexpressor line as previously described^4^). The enzyme solution (1% cellulase R10, 0.25% macerozyme R10, 0.4 M mannitol, 10 mM CaCl_2_, 20 mM KCl, 0.1% BSA, and 20 mM MES, pH 5.7) was used to digest the tape-treated leaf tissue for 3 hours in light (intensity of 50–60 μμmol m^−2^ s^−1^). Following incubation, the pelleted protoplasts were washed twice in chilled W5 solution (154 nM NaCl, 125 mM CaCl_2_, 5 mM KCl, 2 mM MES, pH 5.7) to a density of 2 × 105/mL and incubated on ice for 30 min. The W5 solution was then replaced with MMg solution to a density of 2 × 105/mL.^30^ The protoplasts were transfected by gently mixing 200 μL of protoplasts (2 × 10^5^/mL) in MMg solution (0.4 M mannitol, 15 mM MgCl_2_, 4 mM MES, pH 5.7) with 20 μL of plasmid DNA solution (containing ~30 μg of each construct, dissolved in water), and 220 μL of PEG solution (40% PEG, 0.2 M mannitol, 100 mM CaCl_2_). After incubating at room temperature (RT) for 5 min, the transfection step was stopped by adding 1 mL W5 solution. The protoplasts were spun down at 100 × *g* for 2 min, and the pelleted protoplasts were washed twice in 1 mL W5 solution (0.5 M mannitol, 20 mM KCl, 4 mM MES, pH 5.7). The transfected protoplasts were incubated in the light (intensity of 50–60 μmol m^−2^ s^−1^) at RT for 16–18 hours before being examined using a Leica TCS SP8 laser scanning confocal microscope at the Biological Electron Microscope Facility (University of Hawaii, Honolulu, HI). The excitation/emission filters utilized for fluorescence detection were 488/505–525 nm for GFP (S65T) and 543/585–615 nm for mCherry. For chemical induction of ER stress and the UPR in protoplasts, samples were treated with the chemical ER-stress inducer, 2 mM DTT, from a 1 M DTT stock (Roche Life Science, Inc., Indianapolis, IN) and incubated for 3 hours prior to visualization by scanning confocal microscopy.^25^

## Total fluorescence quantification of protoplast cells

To quantify total fluorescence levels in *Arabidopsis* protoplasts from each genotype transiently expressing the 35S::*bZIP60* intron:GFP construct (with and without 2 mM DTT treatment), cells were imaged on a single plane at 40X magnification (512 by 512 frame resolution) using a Leica TCS SP8 laser scanning confocal microscope. All images were imported as Tif files in ImageJ software (NIH) for quantification of total cell fluorescence. Individual cells were selected and outlined using the ‘freehand selection’ tool and measured for area, integrated density, and mean gray value. Background intensity values were measured by selecting five random areas in the image that do not contain cell fluorescence. Corrected total cell fluorescence (CTCF) values were calculated for each cell using the following equation: CTCF = Integrated intensity – (Area of cell × Mean fluorescence of background readings). Approximately 30–50 cells were analyzed for each sample, and average GFP lifetime values were obtained over two independent experiments.

## RT-PCR of mRNA from leaf mesophyll protoplasts

To analyze transcript levels of UPR marker genes in the WT and OE genotypes, RT-PCR was done on RNA from protoplasts using a protocol as adapted.^25^ Total RNA from 4 week old *Arabidopsis* protoplasts was extracted using the NucleoSpin RNA Plant, Mini kit (Macherey-Nagel, Duren, Germany). Protoplasts from each genotype were resuspended in RAP lysis buffer with mercaptoethanol (1% v/v) and immediately vortexed to mix. Respective samples treated with 2 mM DTT were incubated for 3 hours prior to harvesting of cells and subsequent RNA extraction. cDNA was synthesized using 1 μg of total RNA with M-MLV reverse transcriptase and oligo dT (Promega, Co. Madison, WI). RT-PCR was done using MyFi™ DNA polymerase (Bioline). Primers used for amplification of *PDI9* (PDI9-F, PDI9-R), *bZIP60t* (bZIP60-F, bZIP60-R), *bZIP60s* (bZIP60-F, bZIP60s-R), and *Actin* (Actin-F, Actin-R) transcripts are listed in Table 1. PCR was conducted for 28 cycles. PCR products were resolved by electrophoresis. Relative band intensities were calculated using ImageJ (http://rsb.info.nih.gov/ij/) and normalized to housekeeping gene *Actin* over three replicate cycles.

**Table 1.** List of primers used in this study.

Primer	Sequence, 5’ to 3’
PDI9-F	ACAGGATCCATCTTCGTCACCTGTGGTTCAG
PDI9-R	TCTGCGGCCGCCTTGGATTCTTCACAACTCATC
bZIP60-F	GAAGGAGACGATGATGCTGTGGCT
bZIP60-R	GCAAATGAAGTTTACTCCCAGAAGCCAAAGCAGG
bZIP60s-R	AGCAGGGAACCCAACAGCAGACTC
Actin-F	TCCTTGTACGCCAGTGGTCG
Actin-R	CCGCTCTGCTGTTGTGGTGA

## Results and discussion

**Figure 2.**
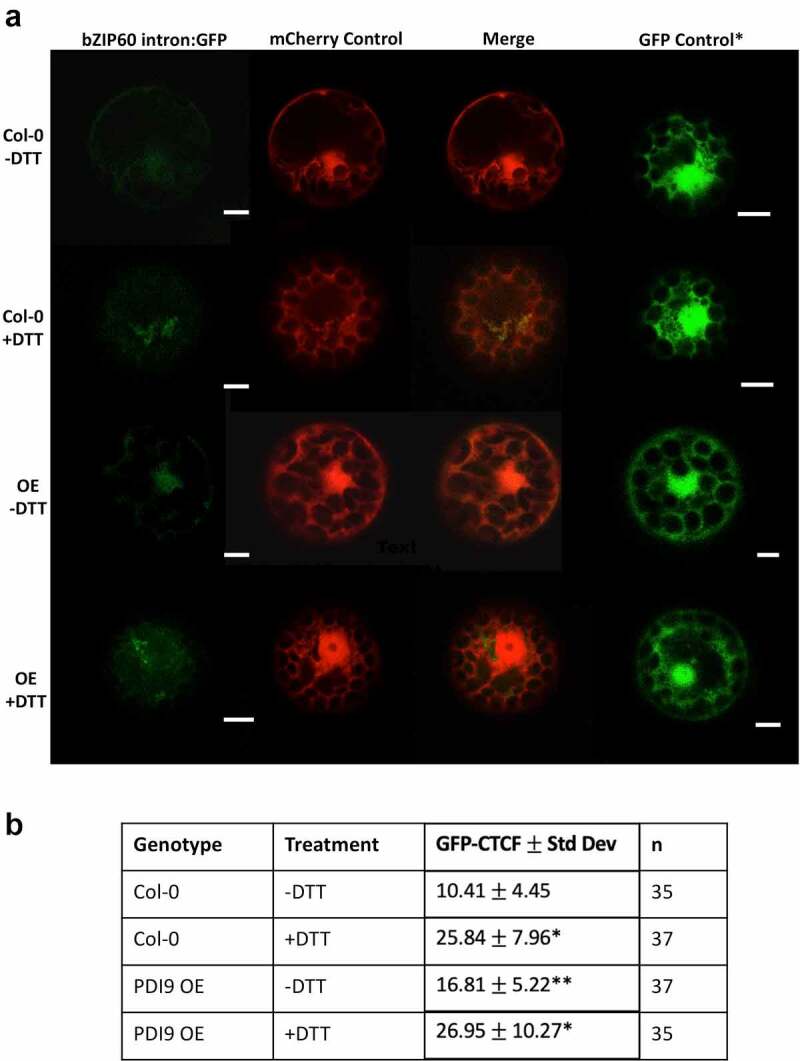
DTT induces and PDI9 modulates splicing of the *bZIP60* intron in *Arabidopsis* protoplasts. a) Representative leaf mesophyll protoplasts transiently expressing the 35S::*bZIP60* intron:GFP reporter construct under normal (-DTT) and ER stressed (+DTT) conditions in two genotypes: wild type (Col-0) and the PDI9 OE. Protoplasts co-transfected with the 35S::mCherry served as a control to assess transfection efficiencies between cells. The GFP, mCherry, and a merge of the two channels are shown. A representative separate independent cell from a single-transfection with the GFP control vector is also illustrated (GFP control) showing cytoplasmic accumulation of GFP. Scale bars are indicated at 5 microns, μ μm. b) Corrected total cell fluorescence (CTCF) values in protoplasts expressing the 35S::*bZIP60* intron:GFP reporter (untreated, -DTT and treated, +DTT). GFP fluorescence was observed by scanning confocal microscopy, and GFP CTCF values were calculated using ImageJ. Showing statistical difference (p < .001) from one-way ANOVA and Tukey honest significant difference (HSD) post-hoc test between samples expressing 35S::*bZIP60* intron:GFP in wild type (Col-0), and OE protoplast cells. Significance with respect to untreated protoplast cells within a genotype group is designated by a single asterisk (*), and significance between genotypes are designated by two (**).

**Figure 3.**
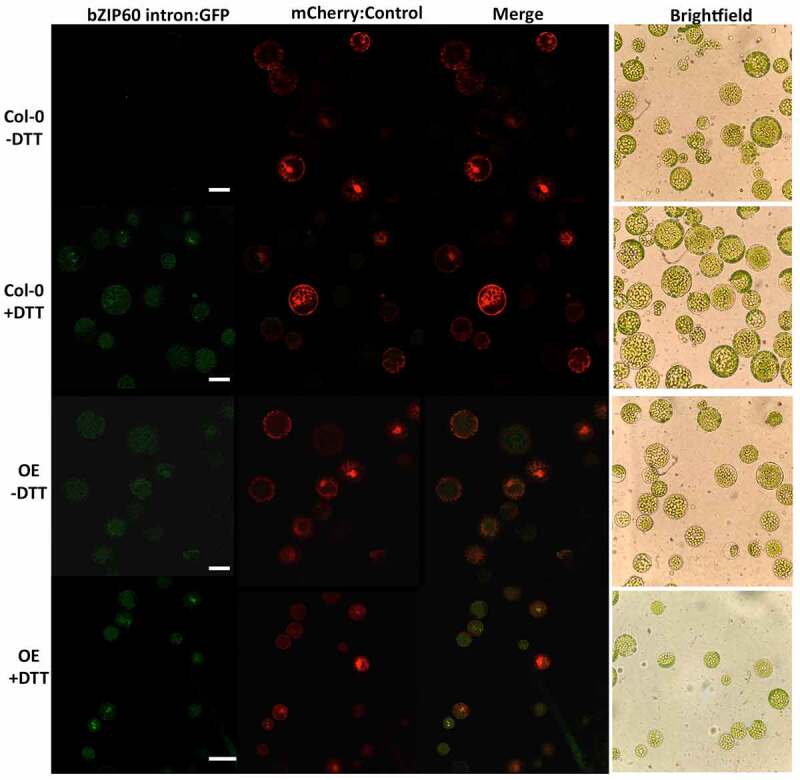
Representative leaf mesophyll protoplasts transiently expressing the 35S::*bZIP60* intron:GFP reporter construct under normal (-DTT) and ER stressed (+DTT) conditions in two genotypes: wild type (Col-0) and the PDI9 OE under the 40× objective at a zoom factor of 1×. The GFP, mCherry, and a merge of the two channels are shown, as well as a corresponding brightfield image of the protoplast cells. Scale bars are indicated at 25 microns, μ μfm.

To develop a UPR biosensor using GFP as the signal output marker, we focused on analyzing the differences in the splicing of the *bZIP60* intron in cells of two different genotypes (WT and PDI9-overexpressor plants) exposed to ER-stress inducing chemical environments. The 35S::*bZIP60* intron:GFP reporter construct was designed with the IRE1-targeted *bZIP60* intronic region, including the key flanking region, fused to the N-terminus of the GFP coding sequence. Splicing of the 23-bp intron (CTCTTGTTGGAATCCCTGCTGTT) causes a frameshift in line with GFP and subsequent formation of ORF2, which has been previously indicated as the natural endogenous frameshift that occurs in *bZIP60* mRNA (Figure 1).^11^ Translation of the unspliced transcript introduces a premature stop codon (UGA) at the N-terminus of GFP, preventing expression (denoted by an *, Figure 1). Therefore, fluorescence as an indicator of GFP expression was measured in transient expression assays of the 35S::*bZIP60* intron:GFP reporter construct in protoplasts from leaves of WT and PDI9-overexpressor plants. In addition, these protoplasts were exposed to the disulfide reducing agent, DTT, which is a chemical inducer of ER stress and UPR (Figures 2, 3).

In mesophyll protoplasts from the WT Col-0 background, the 35S::*bZIP60* intron:GFP reporter is significantly upregulated over twofold in the DTT treatments (Figure 2(a, b)) relative to untreated controls. In untreated protoplasts from the PDI9 overexpressor, there is a 60% increase in GFP fluorescence (Figure 2(a, b)), indicating an increase in UPR in the overexpressor as previously observed in UPR-marker expression analysis in whole plants.^4,9^ Moreover, upon treatment of the 35S::*bZIP60* intron:GFP reporter-containing protoplasts from the PDI9 overexpressor with DTT, the GFP fluorescence increases 60% further relative to untreated control (Figure 2(a, b), Figure 3), indicating enhanced splicing of the *bZIP60* intron from GFP mRNA during DTT-induced ER stress. There was no observable difference in the fluorescence levels of the 35S::mCherry control when co-transfected with the 35S::bZIP60 intron:GFP reporter in WT and OE protoplasts, or when treated with DTT (Figures 2, 3). In both genetic backgrounds, GFP fluorescence intensities were upregulated following DTT treatment and the resulting increase in UPR. We also observed expression and cytoplasmic accumulation of the GFP control in both WT and OE cells under both treated and untreated conditions at levels consistent with that previously reported (Figure 2).^27^ We believe that it would be extremely unlikely that the mRNA encoding GFP alone would bind to, or be spliced by IRE1. However, it is possible that the 35S::*bZIP60* intron:GFP mRNA may compete with the native *bZIP60* mRNA for splicing by IRE1. To our understanding, there are no data in plants suggesting that mechanisms involved in recruitment of *bZIP60* mRNA to IRE1 could be disrupted when fused to GFP. The analogous mammalian^31^ and yeast^32^ models indicate no such disruption.

**Figure 4.**
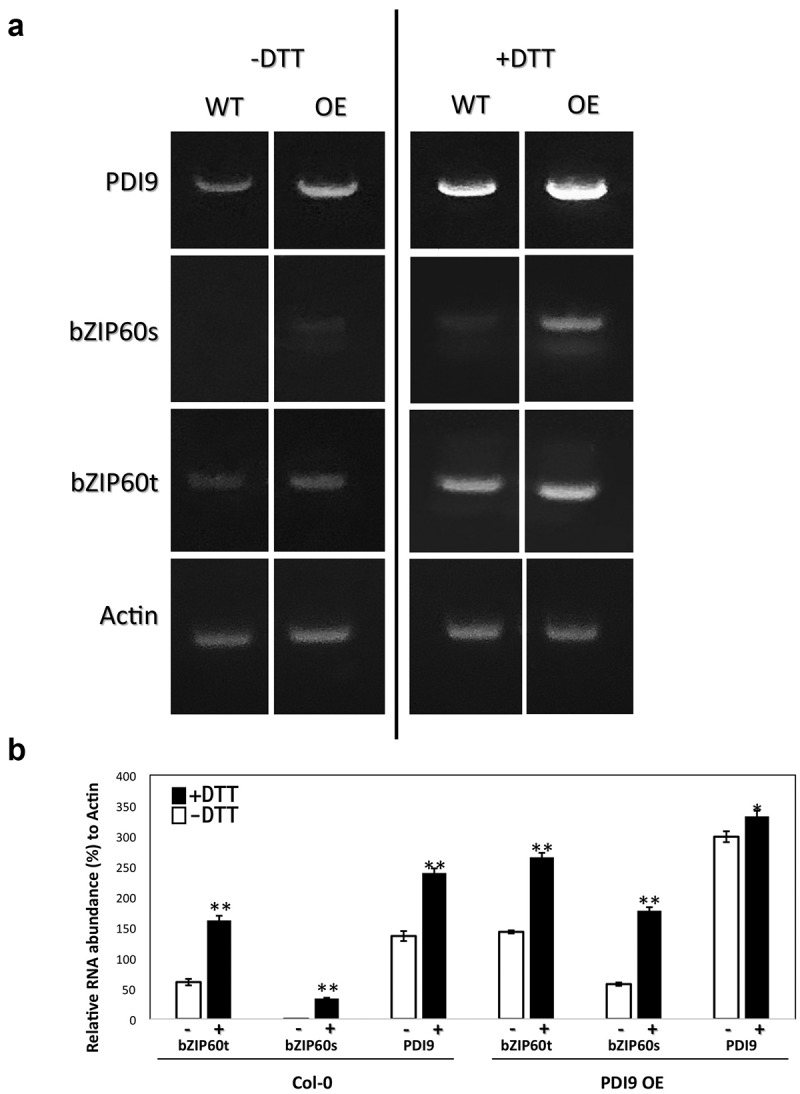
a) A representative agarose gel (0.8% w/v) of resolved RT-PCR products from Col-0 WT and the PDI9 overexpressor (OE) plants following amplification of the transcripts for the *PDI9* gene, the *bZIP60* locus (total RNA = *bZIP60t*, and spliced RNA = *bZIP60s*), and the house keeping gene *Actin* in response to 2 mM DTT treatment in leaf protoplasts. b) Measurement of the RNA levels for the PDI9 gene and the *bZIP60* locus (total RNA = *bZIP60t*, and spliced RNA = *bZIP60s*) in response to 2 mM DTT treatment in leaf protoplasts from Col WT and the OE plants. RT-PCR was conducted and the resulting bands were quantitated relative to actin mRNA controls. The means ± standard deviations are represented from triplicate experiments. **p < .01 and *p < .05, showing statistical difference from one-way ANOVA analysis with post-hoc Tukey HSD Test.

As controls, the mRNA levels for the *PDI9* and *bZIP60* loci were measured via RT-PCR in these same protoplast genotypes and treatments (Figure 4). For the *bZIP60* RNAs, emphasis was placed on distinguishing between total *bZIP60* RNA levels and the spliced *bZIP60* mRNA levels. Two trends were observed. DTT treatment significantly induced the levels of PDI9 and *bZIP60* RNAs (spliced and unspliced) in the WT protoplasts. Likewise, in the PDI9 overexpressor protoplasts, DTT induced the *bZIP60* RNA levels (total and spliced) also by nearly two-fold. Furthermore, in the untreated PDI9 overexpressor protoplasts, *bZIP60* RNA levels (total and spliced) increased over twofold relative to WT, indicating the positive effect PDI9 has on UPR as previously observed.^4^ We conclude that PDI9 increases the UPR as observed via *bZIP60* intron splicing by the IRE1 RNase activity under chemically induced ER stress. In support of these findings, recent data suggest a protective yet modulatory role of PDI9 in pollen development under heat-induced ER stress, in which severe pollen defects were observed in the *pdi9* mutants. Interestingly, a partial phenotype was also observed in the PDI9 overexpressor, suggesting that PDI9 plays an important role in maintaining homeostasis under ER stress. Disrupting the balance of PDI isoforms within the ER may induce an unfavorable condition to maintain proteostasis.^4^

In conclusion, these results support the use of leaf protoplasts and the 35S::*bZIP60* intron:GFP reporter as an experimental system to rapidly monitor UPR via observation and quantification by confocal or fluorescence microscopy. Similar constructs have also been developed in mammalian models to study UPR dynamics.^26,33^ However, no such method has been prior demonstrated in plants, and the utilization of the 35S::*bZIP60* intron:GFP reporter following transient transfection of protoplast cells provides a rapid and reliable method for studying UPR dynamics in plants. The types of treatments can be expanded to include other chemicals or hormones, heat and pathogens, as well as other genes potentially impacting the UPR signaling pathway. The development of such a biosensor also highlights its potential applications in characterizing *IRE1* mRNA targets as a component of RIDD (regulated IRE1-dependent decay), which functions to degrade mRNA-encoding proteins and reduce the secretory load in UPR. Although RIDD has been shown in plants, the identification of substrates and the mechanisms of such degradation are not well understood.^34^ This reporter can also be applied to assess the effects of UPR mutants (i.e. in the ERAD pathway) on affecting the IRE1-based splicing pathway. Finally, by utilizing this reporter, it may be possible to characterize diverse IRE1-target substrates to better understand the nature of how and when IRE1 may degrade specific mRNA targets. The results further reinforce the use of the 35S::*bZIP60* intron:GFP reporter, by expanding its application in transgenic plants, such as crop species, to monitor UPR in the field via multispectral and wavelength-specific drone surveillance.^35^
